# Developmental patterning of sub-epidermal cells in the outer integument of Arabidopsis seeds

**DOI:** 10.1371/journal.pone.0188148

**Published:** 2017-11-15

**Authors:** Elisa Fiume, Olivier Coen, Wenjia Xu, Loïc Lepiniec, Enrico Magnani

**Affiliations:** 1 Institut Jean-Pierre Bourgin, INRA, AgroParisTech, CNRS, University of Paris-Saclay, Versailles, France; 2 Ecole Doctorale 567 Sciences du Végétal, University Paris-Sud, University of Paris-Saclay, bat 360, Orsay, France; Universidad Miguel Hernández de Elche, SPAIN

## Abstract

The seed, the reproductive unit of angiosperms, is generally protected by the seed coat. The seed coat is made of one or two integuments, each comprising two epidermal cells layers and, in some cases, extra sub-epidermal cell layers. The thickness of the seed-coat affects several aspects of seed biology such as dormancy, germination and mortality. In Arabidopsis, the inner integument displays one or two sub-epidermal cell layers that originate from periclinal cell divisions of the innermost epidermal cell layer. By contrast, the outer integument was considered to be two-cell layered. Here, we show that sub-epidermal chalazal cells grow in between the epidermal outer integument cell layers to create an incomplete three-cell layered outer integument. We found that the MADS box transcription factor TRANSPARENT TESTA 16 represses growth of the chalaza and formation of sub-epidermal outer integument cells. Finally, we demonstrate that sub-epidermal cells of the outer and inner integument respond differently to the repressive mechanism mediated by FERTILIZATION INDEPENDENT SEED Polycomb group proteins and to fertilization signals. Our data suggest that integument cell origin rather than sub-epidermal cell position underlies different responses to fertilization.

## Introduction

Angiosperm seeds are generally protected by the seed coat, which develops from the ovule integuments after fertilization [1]. Angiosperm ovules display three main integument architectures: bitegmic ovules are covered by two integuments, unitegmic ovules carry a single integument, and ategmic ovules lack any integuments. Ovule integuments are either two- or multi-cell layered. Both external integument cell layers originate from the epidermal tissue of the chalaza. By contrast, sub-epidermal cell layers develop from periclinal cell divisions of epidermal integument cells or from sub-epidermal chalazal tissue [2]. Seed coat thickness is considered a stable character [2] and affects seed germination, dormancy and mortality [3–5].

Arabidopsis ovules are bitegmic as they carry an outer (oi) and an inner (ii) integument. The ii consists of two epidermal cell layers (ii1 and ii2) and one or two sub-epidermal cell layers (ii1’ and ii1”) [6, 7]. The development of the ii begins with the formation of the ii primordium from two epidermal chalazal cells (ii initials). One of the two ii initials undergoes a periclinal or oblique cell division to create an apical integument cell followed by cell elongation of ii initial cells. Finally, the ii grows by anticlinal cell divisions of the ii1 and ii2 initials to surround the nucellus and female gametophyte [6]. ii1’ and ii1” originate instead by periclinal cell divisions of the ii1 and ii1’, respectively, during the last stage of ovule development [6–8]. Compared to the ii, the oi primordium is larger as it encompasses five to six enlarged epidermal chalazal cells subtended by sub-epidermal chalazal tissue undergoing periclinal cell divisions. Nevertheless, the distal cells of the oi primordium follows the ontogeny of the ii primordium to develop the oi [6]. Sub-epidermal cells have never been observed in the oi, which is considered to be two-cell layered (oi1 and oi2) and solely of epidermal origin [6].

Fertilization triggers the differentiation of ovule integuments into seed coat. Such a developmental shift is repressed by a subset of FERTILZATION INDEPENDENT SEED (FIS) Polycomb group (PcG) proteins. Mutations in the *FERTILIZATION INDEPENDENT ENDOSPERM* (*FIE*) and *MULTICOPY SUPPRESSOR OF IRA1* (*MSI1*) *FIS* genes lead to expansion and differentiation of the epidermal integument cell layers in the absence of fertilization [9, 10]. Fertilization relieves the FIS mediated repression mechanism through a signaling pathway that has been partially elucidated. Kasahara and coworkers have used the *generative cell-specific 1* pollen tube mutant, which undergoes pollen tube rupture but not fertilization, to demonstrate that seed coat differentiation is first triggered by pollen tube content [11]. Exclusive fertilization of the egg or central cell by *kokopelli* mutant pollen has instead highlighted the role of the endosperm in coordinating seed coat development with limited embryo contribution [9, 10]. The endosperm transmits a signal to the seed coat through the action of the AGAMOUS-LIKE 62 (AGL62) MADS box-transcription factor [9, 10]. AGL62 has been proposed to regulate auxin flux, ultimately responsible to induce seed coat differentiation in coordination with endosperm development [12]. The signaling pathway described above applies to the development of the epidermal integument cell layers. By contrast, the endosperm does not initiate the expansion of sub-epidermal ii1’ and ii1” cells in the absence of embryo development. Furthermore, sub-epidermal ii cell layers do not respond to the FIE and MSI1 repressive mechanism [7]. Morphological analyses of the *agl62* seed coat suggest that ii1’ and ii1” cell expansion requires both embryo and endosperm development [13]. All together, these data indicate that growth of seed epidermal and sub-epidermal integument cell layers is regulated by different signaling pathways. It is not known if the unique response to fertilization of the sub-epidermal ii cell layers is due to their sub-epidermal position or to their origin from periclinal cell divisions.

Here, we show that the Arabidopsis oi displays sub-epidermal cell stripes that were previously unnoted and that we refer to as oi’. We followed oi’ cell patterning during ovule development and we discovered that it develops from chalazal sub-epidermal tissue. We identified the MADS box transcription factor TRANSPARENT TESTA 16 (TT16), master regulator of ii1, ii1’ and nucellus development [7, 10, 14–16], as a repressor of chalaza growth. Compared to wild type, *tt16* mutant seeds displayed a longer chalaza along the proximal-distal axis and develop oi’ at a higher frequency. After fertilization, oi’ cells underwent cell expansion. Our data revealed that oi’ expansion is repressed by FIS PcG proteins and is initiated by endosperm growth, as for epidermal integument cell layers. These data suggest that cell origin rather than sub-epidermal cell position influence responsiveness to fertilization.

## Materials and methods

### Plant and genetic materials

*Arabidopsis thaliana* plants, ecotype Columbia (Col) or Wassilewskija (Ws), were used as wild-type controls when appropriate. *kpl-1*, and *tt16-3* lines are in the Ws accession [14, 17]. *goa-1*, *fie-12/+* and *msi1-1/+*, lines are in the Col accession [9, 18, 19]. The *tt16-1* mutant was isolated in the Ws accession and then backcrossed to the Col accession more than three times [10, 14]. Col and Ws *tt16-1* mutants were used according to the experiment. *tt16-1;goa-1* double mutant was generated in the Col accession [19]. Days after flowering (DAF) have been counted starting from the emergence of the pistil from closed flowers [10]. Both DAF and embryo development have been used to determine seed developmental stages.

### Cloning

The *GOA* 1.7 kb promoter and genomic sequence was PCR amplified without the stop codon using the *attB1*-(5’-GCATGAGCTGAGACGCAATC-3’) forward and *attB2*-(5’-AGGAGGTGAAGAACGTCGGTGGGTT-3’) reverse primers. The PCR amplification was performed using the *attB1* (5′-GGGGACAAGTTTGTACAAAAAAGCAGGCT-3′) and *attB2* (5′-GGGGACCACTTTGTACAAGAAAGCTGGGTC-3′) GATEWAY recombination sites at the 5′-ends of the forward and reverse primers, respectively. The PCR product was amplified by high-fidelity Phusion DNA polymerase (Thermo Fisher Scientific Inc.), recombined into the pDONR207 vector (BP Gateway reaction) according to the manufacturer’s instructions (Thermo Fisher Scientific Inc.), and sequenced. The PCR product cloned into the DONR vector was then recombined into the *pMDC107* binary vector [20] (LR Gateway reaction) according to the manufacturer’s instructions (Thermo Fisher Scientific Inc.).

### Transgenic plants

The *Agrobacterium tumefaciens* strain C58C1 was used to stably transform *Arabidopsis* plants through the floral dip method [21]. Transformants were selected by the appropriate resistance and then checked by PCR assays. More than 20 independent transgenic lines were tested for each construct transformed.

### Pseudo-Schiff propidium iodide staining

This protocol allows the staining of cell walls of fixed plant material [10]. More than 20 independent seeds or ovules were analyzed for each genotype and time point.

### Microscopy

mPS-PI and GFP fluorescent imaging was conducted with a Leica TCS-SP5 spectral confocal laser scanning microscope (Leica Microsystems).

### Accession numbers

FIE (AT3G20740), GOA (AT1G31140), KPL (AT5G63720), MSI1 (AT5G58230), TT16 (AT5G23260).

## Results

### Sub-epidermal chalazal cells develop into the oi’

**Fig 1:**
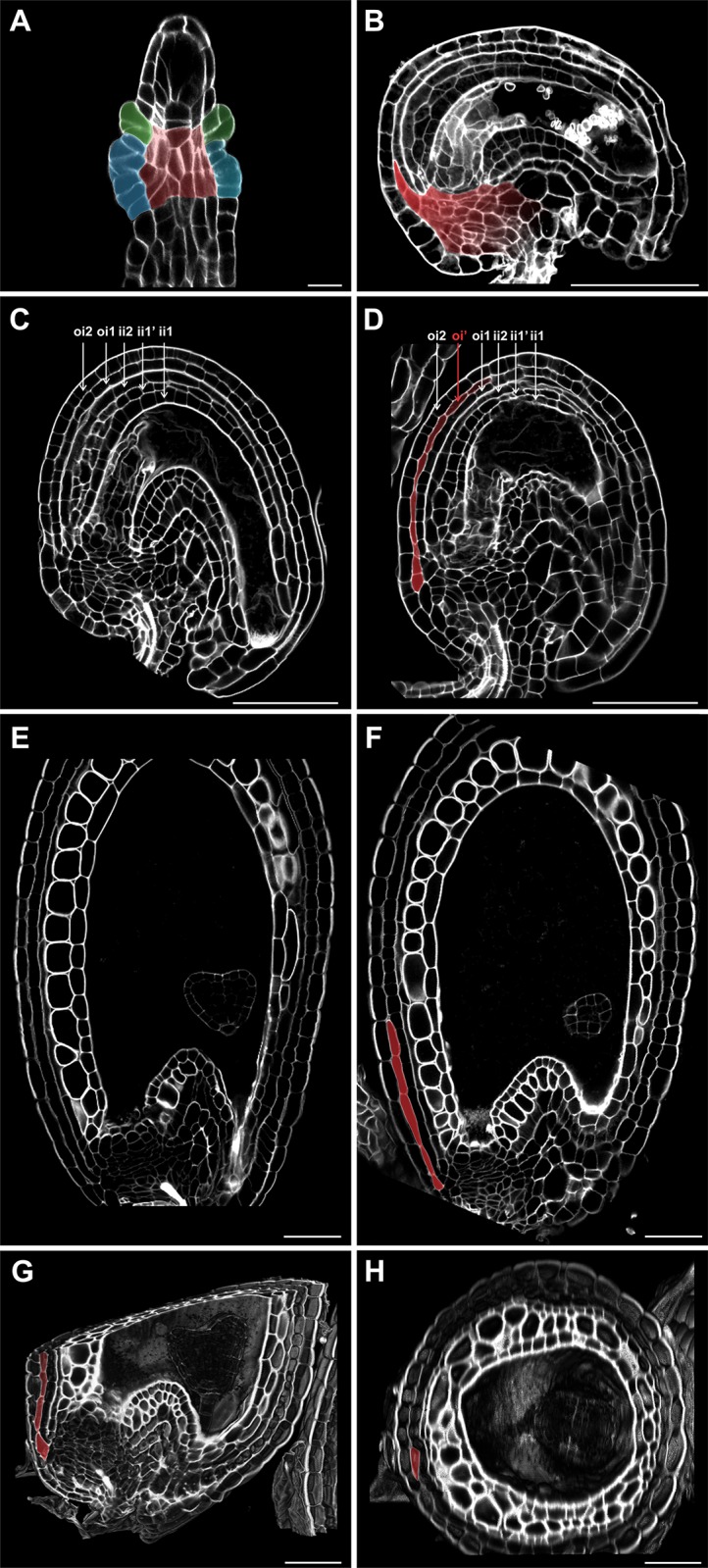
oi’ formation. **(A-B)** Longitudinal sections of wild type ovules at stage 2-III (A) and 3-VI (B) imaged using the mPS-PI technique. Ecotype Col. ii primordium, oi primordium and chalaza are highlighted in green, blue, and red, respectively. Highlighting of the chalazal tissue in red is inferred by the position of the integument primordia. **(C-D)** Longitudinal sections of wild type mature ovules imaged using the mPS-PI technique. Ecotype Col. oi’ is highlighted in red. **(E-F)** Longitudinal sections of wild type seeds at 6 DAF imaged using the mPS-PI technique. Ecotype Col. oi’ is highlighted in red. **(G-H)** Three-dimensional longitudinal (G) and transverse (H) sections of a wild type seed at 6 DAF imaged using the mPS-PI technique. Ecotype Ws. oi’ is highlighted in red. Scale bars, 10 μm (A) and 50 μm (B-H).

The development of Arabidopsis inner and outer integument (ii and oi) primordia is remarkably different. Whereas the ii primordium is compact and comprises three cells (one apical and two initial cells), the oi primordium extends to cover the entire proximal region of the chalaza and its convexity is partially due to periclinal cell divisions of sub-epidermal chalazal cells (Fig 1A). Nevertheless, the distal part of the oi primordium has been described to develop as a two-cell layered tubular sheet, similarly to the ii [6]. To better analyze oi development, we analyzed longitudinal sections of Arabidopsis ovules three dimensionally reconstructed using the modified pseudo-Schiff propidium iodide imaging technique (mPS-PI, see materials and methods) [10]. At stage 3-VI of ovule development, we detected one to three sub-epidermal chalazal cells intercalating into the proximal oi1 and oi2 in 62% of the ovules tested (n = 212, Col accession) (Fig 1B). In mature ovules and in seeds, we identified longer stripes of sub-epidermal oi cells (> 3 cells) that we named oi’ (Fig 1D and 1F). In most extreme cases, the oi’ reached up to the bending zone (Fig 1D). 10.1% of seeds (n = 234, Col accession) at 6 days after flowering (DAF, see materials and methods) displayed an oi’ (Fig 1D and 1F compared to 1C and 1E), indicating that not all oi’ initial cells divide further along the proximal-distal axis.

Images of oi’ cells three dimensionally reconstructed using the mPS-PI technique appeared tubular in shape and oriented along the proximal-distal axis (Fig 1G). Furthermore, we detected up to 3 adjacent oi’ cell stripes (Fig 1H) but never a oi’ cell layer covering the entire seed surface as it is for the other oi cell layers.

### oi’ formation correlates with chalazal growth

**Fig 2:**
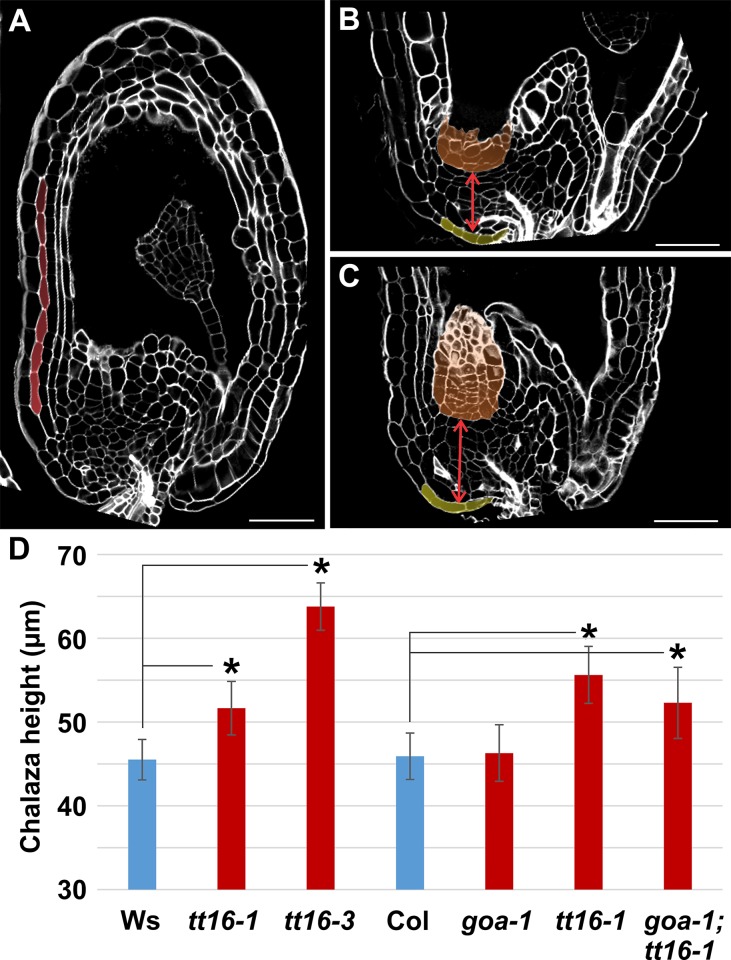
Growth of the chalazal tissue promotes oi’ formation. **(A-C)** Longitudinal sections of *tt16-1* (A), wild type (B), and *tt16-3* (C) seeds at 6 DAF imaged using the mPS-PI technique. Ecotype Ws. oi’, nucellus, and proximal oi are highlighted in red, orange and yellow, respectively. Red arrows indicate the chalaza height. **(D)** Average chalazal height as observed in central longitudinal sections of wild type and mutant seeds imaged using the mPS-PI technique. Chalaza height was measured from the proximal nucellus till the proximal oi as shown in panels A and B (red arrows). Asterisks indicate statistical difference between wild type and mutant lines (two-tailed student's *t* test, P < 0.05). Error bars: standard deviations. n>20.

We noticed that the incidence of oi' formation was higher in the *transparent testa 16* (*tt16*) mutant (22.4%, n = 62 in *tt16-1* and 17.6%, n = 45 in *tt16-3*; Ws accession) compared to wild type (10.3%, n = 263 Ws accession) (Fig 2A). The TT16 MADS box transcription factor regulates several aspects of seed maternal tissue development such as ii1 and ii1’ cell growth, orientation and differentiation and nucellus degeneration [7, 10, 14, 15]. Since the oi’ originates from sub-epidermal chalazal tissue we analyzed the size of the chalaza in wild type and *tt16* mutant seeds. Compared to other seed tissues, not all borders of the chalaza can be identified through morphological markers. Therefore, we measured the height of the chalaza defined as the distance between the nucellus and the proximal oi2, which can be easily recognized in seed longitudinal sections (Fig 2B and 2C, red arrows). The height of the chalaza of both *tt16-1* (n = 22) and *tt16-3* (n = 25) seeds was bigger than that of wild type seeds (n = 73) (Fig 2D). TT16 has been shown to have redundant functions with its closest paralogue GORDITA (GOA) [10]. Therefore, we analyzed chalaza and oi’ development in *goa-1* and *goa-1;tt16-1* mutants. *goa-1* mutation did not affect the height of the chalaza in *TT16* wild type (n = 46) and mutant (n = 27) backgrounds (Fig 2D). Finally, the incidence of oi’ formation in *goa-1* (10%, n = 130 Col accession) and *goa-1;tt16-1* (20.5%, n = 53 Col accession) seeds was comparable to that observed in wild type (10.1%, n = 234 Col accession) and *tt16-1* (21.7%, n = 58 Col accession) seeds, respectively. Altogether, these data suggest that the higher chalazal tissue of *tt16* seeds might favor oi’ formation. Nevertheless, we cannot exclude a direct role of TT16 in oi’ development.

RNA *in situ* hybridization and gene promoter analyses revealed *TT16* expression in ii1, ii1’ and nucellus but not in the chalaza [7, 10]. Nevertheless, a number of genetic evidences showed TT16 non-cell autonomous effect [7, 10]. For example, *TT16* complemented tt16 integument defects when expressed solely in the nucellus and vice versa [10]. Therefore, we hypothesize that TT16 might influence the development of the chalaza in a non-cell autonomous fashion from the nucellus or the ii1. We tested *GOA* expression in seeds carrying *GOA* promoter and genomic regions fused to the gene encoding for the green fluorescent protein (GFP) (S1 Fig). We detected GFP fluorescence in the nuclei of the chalaza. This finding suggests a role for *GOA* in the development of the chalaza or neighboring tissues that is yet to be understood.

### oi’ cell expansion is repressed by FIS PcG proteins and induced by endosperm development

**Fig 3:**
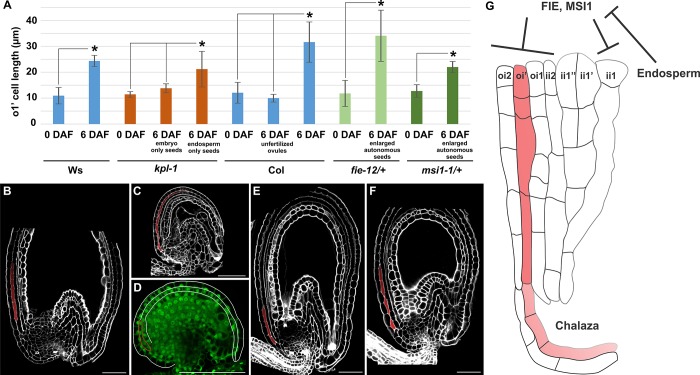
oi’ cell expansion is repressed by FIS PcG proteins and induced by endosperm growth. **(A)** Average length of the most proximal oi’ cell as observed in longitudinal sections of wild type and mutant seeds imaged using the mPS-PI technique. Asterisks indicate statistical difference (two-tailed student's *t* test, P < 0.05). Error bars: standard deviations. n>20. **(B-C)** Longitudinal sections of *kpl-1* only-endosperm (B) and only-embryo (C) seeds at 6 DAF imaged using the mPS-PI technique. Ecotype Ws. **(D)** GFP fluorescence image of a *ProFIE*:*gFIE-GFP* ovule at stage 3-VI. oi1 and oi2 cell layers are marked by white lines whereas oi’ cells are marked by a red line. **(E-F)** Longitudinal sections of *fie12/+* (E) and *msi1-1/+* (F) unfertilized large autonomous seeds at 6 DAF imaged using the mPS-PI technique. Ecotype Col. **(G)** Model for the development of the seed oi’. Black arrows indicate functional relationships. Scale bars, 50 μm. oi’ and chalazal sub-epidermal cells are highlighted in red.

To test if oi’ cells respond to fertilization, we measured their length in ovule and seed longitudinal sections before, after and in the absence of fertilization. Between 0 and 6 DAF, the first proximal oi’ cell more than doubled in length along the proximal-distal axis (Fig 3A). By contrast, oi’ cells of unfertilized ovules did not change in size during the same time period (Fig 3A). These data demonstrate that fertilization triggers oi’ cell expansion, in line with what observed for all other integument cell layers [7, 9].

Whereas epidermal integument cell layers elongate in response to endosperm growth, sub-epidermal ii1’ and ii1” cell layers appear to require both embryo and endosperm development to expand [7, 13]. To study the fertilization signaling pathway underlying oi’ growth, we analyzed seeds of the *kokopelli* (*kpl*) mutant, which displays random single-fertilization events [17]. Seeds that develop only the endosperm (endosperm-only seeds) carry elongated epidermal integument cell layers and unexpanded sub-epidermal ii1’ and ii1’ cells. By contrast, epidermal and sub-epidermal integument cell layers of embryo-only seeds do not expand [7, 9]. As for epidermal integument cell layers, oi’ cells elongated in endosperm-only seeds but not in embryo-only seeds (Fig 3A–3C). The endosperm has been shown to induce the expansion of epidermal integument cell layers by relieving the repression mechanism mediated by FERTILZATION INDEPENDENT SEED (FIS) Polycomb group (PcG) proteins [9]. Therefore, we analyzed oi’ cells in *fertilization independent endosperm* (*fie*) and *multicopy suppressor of ira1* (*msi1*) unfertilized enlarged autonomous seeds, which display elongated epidermal integument cells and unexpanded sub-epidermal ii1’ and ii1” cells [7, 9]. oi’ cells of both *msi1* and *fie* enlarged autonomous seeds underwent cell elongation between 0 and 6 DAF, comparable to that observed in fertilized seeds (Fig 3A, 3E and 3F). *FIE* and *MSI1* are expressed in ovule sporophytic tissues [10, 22, 23] but their expression in oi’ cells has never been tested before. We analyzed *FIE* expression in ovules by using a line carrying *FIE* promoter and genomic sequence fused to *GFP* [23], which recapitulates *FIE* expression as detected by RNA in situ hybridization experiments [10]. We detected GFP fluorescence in all integument cell layers including oi’ (Fig 3D). Overall, these data indicate that oi’ growth is repressed in the ovule by FIE and MSI1 and induced after fertilization by endosperm growth, the same regulatory mechanism underlying the expansion of epidermal integument cell layers.

## Discussion

Integument thickness has been described as a stable character and is used in macro-systematic analyses [2]. Nevertheless, we have recently shown that ii thickness in Arabidopsis changed in 25% of the ovules tested (Col accession) due to the formation of the ii1” cell layer [7]. Here, we showed that sectors of the oi are three-cell thick in 10% of the seeds analyzed because of the growth of sub-epidermal chalazal tissue in between oi1 and oi2. Our data indicate that developmental stability of oi thickness is regulated by TT16. The analysis of the chalazal tissue is difficult due to the absence of clear morphological markers all along its borders and to irregular cell divisions occurring along different planes. Nevertheless, the analysis of *tt16* seeds revealed a correlation between the height of the chalaza and the formation of the oi’. We speculate that physical separation between the proximal oi1 and oi2 cells might influence the formation of oi’ by facilitating the growth of sub-epidermal chalazal cells in between oi1 and oi2. Alternatively, oi’ growth might be influenced by the cell division rate of the chalaza. The evolutionary and environmental implications of such developmental variation are yet to be fully understood. Nevertheless, sub-epidermal ii cell layers have been speculated to offset perturbations in the seed coat developmental program [7]. oi’ cells might play a similar role and compensate for an unbalanced growth of the ovule integuments.

Our *GOA* expression analysis suggests a role for GOA in the development of the chalaza. This hypothesis is strengthened by TT16 (GOA closest paralogue) function in regulating chalaza growth. Nevertheless, *goa* mutation did not affect the length of the chalaza. We speculate that GOA might play other roles in chalaza development. In the future, analyses at the sub-cellular level or a better morphological characterization of the chalaza might shed some light on GOA function.

Finally, our data revealed that sub-epidermal oi and ii cell layers respond to different fertilization signals. oi’ cell expansion is repressed by FIS PcG proteins and induced by endosperm growth, the same response observed in epidermal integument cell layers. By contrast, ii1’ and ii1” cell layers appear to require embryo and endosperm development [7, 13]. This observation discards the hypothesis that the unique response to fertilization of ii1’ and ii1” is due solely to their sub-epidermal position. We speculate that ii1’ and ii1” origin by periclinal cell divisions might account for such a different response to fertilization compared to oi’ and epidermal integument cell layers.

## Supporting information

S1 Fig *GOA* expression. GFP fluorescence image of a *ProGOA*:*gGOA-GFP* ovule at stage 3-VI. The contour of the ovule is marked by a white line. A red arrowhead points to the chalazal nuclei expressing GFP. Scale bars, 50 μm.
(DOCX)
