# Supplementary material for: Developmental patterning of sub-epidermal cells in the outer integument of Arabidopsis seeds
Source: PLoS One. 2017 Nov 15;12(11):e0188148. doi: 10.1371/journal.pone.0188148 (PMC5687734; doi:10.1371/journal.pone.0188148)
Supplement: S1 Fig — GFP fluorescence image of a ProGOA:gGOA-GFP ovule at stage 3-VI. The contour of the ovule is marked by a white line. A red arrowhead points to the chalazal nuclei expressing GFP. Scale bars, 50 μm. (DOCX) [file pone.0188148.s001.docx]

**Supporting information**

**
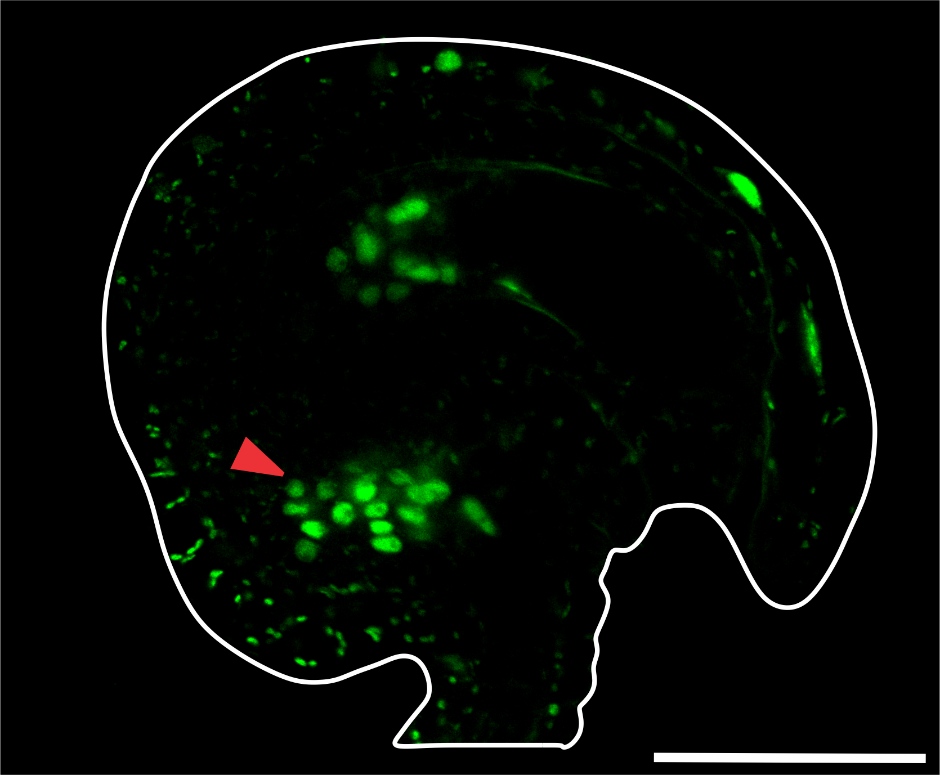
**

**S1 Fig. *GOA* expression**

GFP fluorescence image of a *ProGOA:gGOA-GFP* ovule at stage 3-VI. The contour of the ovule is marked by a white line. A red arrowhead points to the chalazal nuclei expressing GFP. Scale bars, 50 µm.
